# Etats hémodynamiques et respiratoires des opérées de fistules obstétricales sous rachianesthésie au CHRR Manakara, Madagascar

**DOI:** 10.11604/pamj.2016.25.140.10813

**Published:** 2016-11-11

**Authors:** Andrianimaro Florelia Martinetti, Rabenjarison Franklin, Randriamboavonjy Rado Lalao, Harioly Nirina Marie Osé Judicael, Rasolonjatovo Tsiorintsoa Yvonne, Rajaonera Tovohery Andriambelo, Rakotoarison Ratsaraharimanana Catherine Nicole, Raveloson Nasolotsiry Enintsoa, Ravaomanana Edwige

**Affiliations:** 1 Service Bloc opératoire- Réanimation, CHRD Moramanga, Madagascar; 2 Accueil-Triage-Urgences-Réanimation Médicale, Hôpital Joseph Raseta de Befelatanana, CHU Antananarivo Madagascar; 3 Faculté de Médecine d'Antananarivo Madagascar; 4 Service Bloc opératoire-Réanimation, CHU Toamasina Madagascar; 5 Service Bloc opératoire-Réanimation, Chirurgie maxillo-faciale Befelatanana, CHU, Antananarivo, Madagascar; 6 Service Réanimation Chirurgicale, Hôpital Joseph Ravoahangy Andrianavalona, CHU Antananarivo, Madagascar; 7 Accueil-Triage-Urgences-Réanimation, Hôpital Joseph Ravoahangy Andrianavalona, CHU Antananarivo, Madagascar; 8 UNFPA - Madagascar

**Keywords:** hemodynamic, obstetric fistula, respiratory effect, spinal anesthesia, Trendelenburg, Respiratory effects, hemodynamic, obstetric fistula spinal anesthesia, Trendelenburg

## Abstract

L'objectif était d'évaluer les états hémodynamiques et respiratoires des opérées de fistules obstétricales et rapporter nos expériences sur la prise en charge de ces malades. Il s'agit d'une étude transversale descriptive effectuée au Bloc opératoire et Réanimation du CHRR Manakara allant du 20 au 30 aout 2013. Etaient incluses les patientes opérées de fistules obstétricales sous rachianesthésie. Nous avons exclu les patientes classées ASA >2 et celles ayant des tares cardio-vasculaires ou respiratoires. Après consultations préanesthésiques, et bilans préopératoires, nous avons administré chez les patientes 12,5mg de bupivacaïne adrénalinée 0,5% isobare en intrathécal. Elles étaient mises en position de Trendelenburg 5 minutes après l'injection du produit anesthésique et durant toute l'intervention. Le niveau sensitif, la pression artérielle, la fréquence cardiaque, la fréquence respiratoire et la saturation pulsée en oxygène (SpO2) étaient enregistrés pendant l'intervention. Nous avons retenues 57 malades. Le bloc sensitivomoteur était excellent pour toutes les malades. Un niveau métamérique supérieur à T6 était atteint chez 56,36% des malades. A part quelques épisodes d'hypertension artérielle et tachycardie, nous n'avons trouvé aucune difficulté respiratoire ni cardiovasculaire en rapport avec la position. Seuls, le niveau sensitif et la fréquence respiratoire présentaient une corrélation (p=0,01). Cette étude suggère que la position de Trendelenburg est réalisable au cours de la rachianesthésie en utilisant un produit anesthésique approprié, en faisant attention, notamment aux changements de position et en tenant compte de l'examen préanesthésique.

## Abstract (en)

The objective was to evaluate the hemodynamic and respiratory conditions of patients who had undergone obstetric fistula surgery and to report our experience in the management of these patients. We conducted a descriptive cross-sectional study in the Operating Room and Intensive Care Unit of the Regional Reference Hospital in Manakara from 20 to 30 August 2013. All patients who had undergone obstetric fistula surgery under spinal anesthesia were included in the study. We excluded patients classified as ASA> 2 and those with cardiovascular or respiratory disorders. After pre-anesthetic consultation and preoperative assessments, patients received bupivacaine 12.5 mg with adrenaline 0,5% isobar intrathecal. They were placed in the Trendelenburg position for 5 minutes after the injection of anesthesia and during surgery. Intraoperative sensory level, blood pressure, heart rate, respiratory rate and peripheral capillary oxygen saturation (SpO2) were recorded. 57 patients were retained. The sensory and motor block were excellent in all patients. A metameric level higher than T6 was achieved in 56.36% of patients. Despite a few episodes of hypertension and tachycardia, no patient had respiratory or cardiovascular problems associated with Trendelenburg position. Only sensory level and respiratory rate showed a correlation with Trendelenburg position (p=0,01). This study suggests that Trendelenburg position can be used during spinal anesthesia if an appropriate anesthetic is administered, paying particular attention to changes in position and taking into account the pre-anesthetic examination.

## Introduction

La fistule obstétricale (FO) est une affection due à des complications survenues pendant l'accouchement. Son développement est directement lié à la dystocie mécanique. Plus de 2 millions de jeunes femmes vivent avec une FO non soignée, et que 50 à 100 000 nouveaux cas surviennent chaque année [1]. La plupart des FO peuvent être traitées par une intervention chirurgicale simple. Elle est généralement réalisée sous rachianesthésie qui est la technique préférée car c'est simple, fiable, efficace et moindre coût. L'opération en position de lithotomie et Trendelenburg présente un risque théorique de survenue d'hypotension artérielle sévère, de rachianesthésie totale, de difficulté respiratoire et d'arrêt cardiaque par extension du bloc [2]. Nous avons effectué une étude menée du 20 au 30 août 2013 au Centre Hospitalier de Référence Régionale de Manakara Madagascar afin d'évaluer les états hémodynamiques et respiratoires des femmes opérées de fistule obstétricale sous rachianesthésie ainsi que de rapporter nos expériences.

## Méthodes

**Figure 1:**
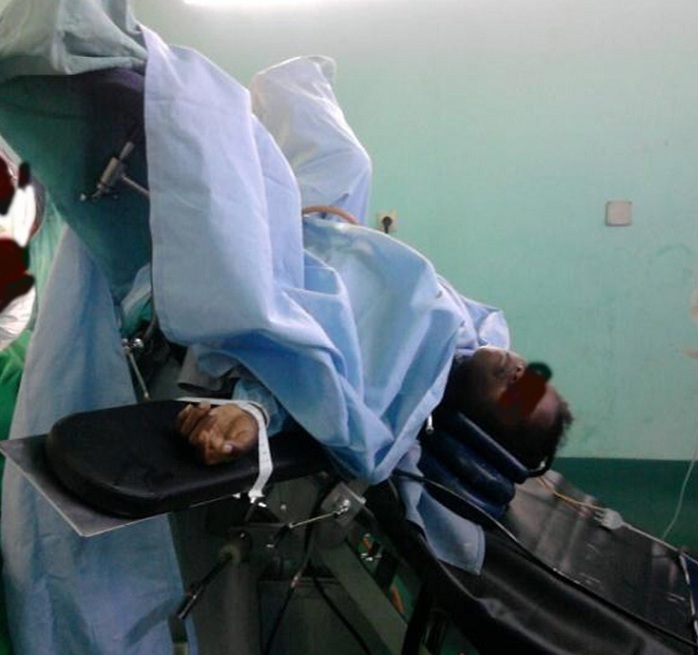
Position des malades durant l'opération (Trendelenburg et Lithotomie)

Il s'agit d'une étude descriptive transversale exhaustive effectuée aux Services de Bloc opératoire et Réanimation du Centre Hospitalier de Référence Régionale Manakara, Madagascar au cours de la campagne de Fistule Obstétricale du 20 au 30 aout 2013 à Manakara. Nous avons inclus les patientes classées ASA 1 et 2 (Classification proposée par the American Society of Anesthesiologists) [3] admises et proposées pour une opération de fistule obstétricale. Les malades présentant des contre-indications de la rachianesthésie et les opérées sous anesthésie générale, ainsi que les malades ayant des antécédents de troubles hémodynamiques (Hypertension artérielle, insuffisance cardiaque,…) et respiratoires (BPCO, asthme…) en cours étaient exclues. La consultation pré-anesthésique et les bilans préopératoires (notamment le test d'Emmel, la numération des formules sanguines, le groupage sanguin, le bilan rénal et la coagulation) étaient systématiques. Toutes les malades ont reçu un préremplissage de 500mL de Ringer lactate. Nous avons administré 12,5 mg de bupivacaïne à 0,5% isobare adrénalinée par voie intrathécale chez les patientes en position assise au niveau de l'espace L4-L5 à l'aide d'une aiguille 22G. Puis elles étaient réinstallées en décubitus dorsal pendant 5 minutes, en position de Trendelenburg (45°) cinq minutes après l'injection du produit anesthésique et durant toute l'intervention. Les caractéristiques du bloc, les paramètres hémodynamiques et respiratoires ont été enregistrés pendant la rachianesthésie, avant et après la mise en position de Trendelenburg (Figure 1).

Les pressions artérielles systoliques et diastoliques ont été enregistrées juste avant la ponction rachidienne, puis toutes les 2 minutes. Nous avons noté les pressions artérielles systoliques (PAS) minimales et maximales. L'hypotension a été définie par une PAS inférieure à 100 mmHg et/ou sa réduction de plus de 30% par rapport aux chiffres contrôles. Cette hypotension a été traitée par des bolus de 10 mg d'éphédrine. La consommation totale d'éphédrine a été relevée. L'hypertension a été définie par une PAS supérieure ou égale à 140 mmHg. Nous avons également enregistré les fréquences cardiaques et les fréquences respiratoires ainsi que la saturation pulsée en oxygène (SpO2) pendant toute l'intervention. L'évaluation du niveau sensitif a été procédée sur la ligne médio claviculaire par le test du pique touche au froid (de haut en bas), toutes les minutes: la ligne mamelonnaire correspond à un bloc qui remonte jusqu'au quatrième métamère thoracique (T4), l'appendice xiphoïde correspond au sixième métamère thoracique (T6), le nombril correspond au dixième métamère thoracique (T10), le pubis au premier métamère lombaire. L'analyse statistique des données a été réalisée avec le logiciel R CRAN. Le test du Khi-carré de Pearson (χ^2^) sera utilisé. Sauf indication contraire, les données présentées sont les moyennes ± Ecart-type (ET). Le seuil de significativité a été fixé à une valeur de p < 0,05.

## Résultats

Après consultation pré-anesthésique, 60 patientes classées ASA1 et ASA2 âgées de 17 à 58 ans, opérées pour fistules obstétricales ont été recrutées. La plupart des lésions fistuleuses étaient classées simples. Nous avons retenu 55 femmes. Cinq malades ont été exclues de l'étude dont 2 hypertendues et 3 autres, opérées sous anesthésie générale. La moyenne d'âge était de 31 ans avec des extrêmes de 17 et 55 ans. Personne n'a présenté, ni obésité, ni tare associée à part les neuf patientes drépanocytaires (Tableau 1). Les paramètres hémodynamiques des patientes étaient relativement stables (Tableau 2). Au départ, c'est-à-dire avant l'infiltration, les pressions artérielles systoliques étaient 131,27 ± 11,06 mmHg en moyenne, avec un minimum de 100 mmHg et un maximum de 150 mmHg. Les patientes ayant des chiffres tensionnels inférieurs à 100 mmHg étaient au nombre de 11 (20%), dont 4 (7,2%) ont eu 70 mmHg. Seize (29%) malades avaient une incidence de chiffres tensionnels supérieurs à 140 mmHg. La survenue d'une bradycardie, c'est-à-dire une fréquence cardiaque inférieure à 50 battements par minute (bpm) était nulle, avec 73,65 ± 8,76 bpm en moyenne pour la fréquence cardiaque minimale. Cependant, 9 (16,36%) malades avaient présenté une tachycardie, c'est-à-dire une fréquence cardiaque supérieure ou égale à 100 bpm. C'est pareil pour les paramètres respiratoires, on n'a décelé aucune incidence de désaturation. Les valeurs minimales de la saturation pulsée en oxygène varient entre 95 et 99% avec 96,67± 0,88% en moyenne (Tableau 2). Sauf que 16 (29%) malades avaient une incidence de fréquence respiratoire supérieure à 20 cpm et la différence entre les fréquences respiratoires maximales et minimales était significative (p=0,04). Le niveau métamérique T4 a été atteint chez 31 (56,36%) malades et un niveau T6 pour le reste. Seul le niveau sensitif présente une relation significative avec la fréquence respiratoire (p=0,01). Plus le niveau est supérieur et plus la fréquence respiratoire s'accroit. Par contre, ni l'âge, ni l'indice de masse corporel (IMC), ni le test d'Emmel n'influent sur l'augmentation de la fréquence respiratoire avec des valeurs de p respectivement égales à 0,48 ; 0,52 et 0,88. En terme de consommation en éphédrine, 11 (20%) malades en avaient besoin pendant l'intervention et la dose moyenne était de 6,98 ± 3,96 mg. Mais elle n'a pas de relation, ni avec l'âge, ni avec l'IMC, ni avec le test d'Emmel, ni avec le niveau sensitif avec des valeurs de p respectivement égales à 0,93 ; 0,21 ; 0,33 et 0,91.

**Tableau 1:** Caractéristique et classification des patientes

Paramètres	Moyenne ± ET	Minimum	Maximum	n (%)
Age (ans)	31,09 ±9,99	17	55	
IMC	20 ±1,51	17	23	
Parité (n)	2,56 ±2,65	1	11	
ASA				
1				45 (81,82)
2				10 (18,18)
Tares associées				
Drépanocytose				9 (16,36)
Autres				0

ASA: American Society of Anesthesiology - ET: écart-type - IMC: Indice de Masse corporelle

**Tableau 2:** Les paramètres hémodynamiques et respiratoires

	Contrôle	Minimum	Maximum	p-value
	Moyenne ±ET (Min- Max)	Moyenne ±ET (Min- Max)	Moyenne ±ET (Min- Max)	
**Paramètres hémodynamiques**				
PAS (mmHg)	131 ,27±11,06(100-150)	102,72±14,33(70-140)	139,27±12,74(110-170)	0,1
Fréquence Cardiaque (bpm)	-	73,65±8,76(55-90)	74±9,48(74-115)	0,35
**paramètres respiratoires**				
Fréquence Respiratoire (cpm)	-	15,89±1,83(14-20)	20±2,48(16-28)	0,04
SpO2 (%)	-	96,67±0,88(95-99)	99,25±0,67(98-100)	0,25

bpm: battement par minute – cpm : Cycles par minute – ET: Ecart type – max: maximale - min: minimale - PAS : pressions artérielles systoliques – SpO2 : Saturation pulsée en oxygène

## Discussion

A Madagascar, 2.000 nouveaux cas de FO par an sont décelés, soit 2 à 3 cas pour 1 000 grossesses. De ce fait, des activités préconisées pour l'éradication des fistules obstétricales et d'améliorer la qualité de la prise en charge des femmes porteuses de fistules ont été planifiées et mises en œuvre dans quelques centres hospitaliers de référence [4]. L'opération est habituellement réalisée sous rachianesthésie et en position de Trendelenburg avec lithotomie. Les états hémodynamiques et respiratoires des malades opérées sont rarement étudiés. Pourtant, au cours d'une rachianesthésie, l'hypotension survient plus rapidement, et est majorée parce que la vitesse d'installation du bloc est supérieure à celle de la mise en jeu des phénomènes de compensation physiologiques. Elle est principalement responsable d'une vasodilatation artérielle et d'un pooling veineux [5]. Les effets hémodynamiques d'une mise en Trendelenburg ou de lithotomie sont d'abord une augmentation du débit cardiaque consécutive à un accroissement du volume sanguin thoracique, par redistribution aux dépens des secteurs splanchniques et des membres inférieurs. En pratique, les phénomènes de redistribution interne sont limités à partir d'une position de décubitus [6, 7], le volume sanguin central n'augmentant que de 1,8% après mise en position de Trendelenburg à 15°. Pour des inclinaisons supérieures à 20°, l'engorgement des vaisseaux thoraciques et le poids des viscères sur le diaphragme, en augmentant la pression intra thoracique, réduisent l'efficacité du gradient de remplissage entre la veine cave inférieure et l'oreillette droite, et pourraient ainsi diminuer le débit cardiaque [8]. Ainsi, dans une étude qui consistait à augmenter le niveau du bloc d'une rachianesthésie, Kim JT et al ont mis en position de Trendelenburg 2 groupes de patients (l'un en Trendelenburg simple et l'autre avec position de lithotomie). La pression artérielle moyenne et la fréquence cardiaque ont été diminuées dans le groupe avec position de lithotomie, et la consommation en éphédrine est augmentée par rapport à l'autre groupe. Chez ce même groupe, le niveau maximal du bloc sensitif est plus élevé. Le bloc moteur n'était pas différent entre les 2 groupes. Il faut noter qu'un malade a présenté une dyspnée associée à une extension du bloc supérieur au niveau T4 [9]. Ponhold et al ont administré 4 ml de bupivacaïne 0,5% en intrathécale chez trois groupes de 20 patients mis en décubitus dorsal, en Trendelenburg à 30° et en hamac (tronc et les jambes élevés à 30 °). L'incidence de bradycardie grave (FC<50bpm) était significativement plus élevée chez les patients en position de Trendelenburg (60%) que ceux en position horizontale (20%, p <0,01) ou en hamac (10%, p<0,005) [10].

En position de lithotomie, les perturbations hémodynamiques peuvent être importantes lors du repositionnement des membres inférieurs à l'horizontale, avec risque de collapsus par séquestration sanguine [11]. Selon l'étendue du bloc sympathique, les anesthésies locorégionales rachidiennes exposent au même risque, pour des inclinaisons moindres [12]. Nishikawa et al ont rapporté le cas d'une bradycardie progressive suivie d'un arrêt cardiaque lors d'une anesthésie rachidienne chez une patiente âgée de 26 ans survenant immédiatement après changement de position de Trendelenburg de 15° à la position horizontale [13]. Dans notre étude où les malades étaient mises en Trendelenburg avec position de lithotomie, les épisodes d'apparition d'hypotension artérielle existaient mais ils étaient bien moindres (au nombre de 11 ou 20%, dont 4 ou 7,2% ont eu 70 mmHg) par rapport aux survenues d'hypertension artérielle (supérieure à 140 mmHg) qui était au nombre de 16 (29%). L'incidence de bradycardie était quasiment nulle avec une fréquence cardiaque minimale de 55 bpm. Par contre, neuf malades (16,36%) avaient présenté une tachycardie, c'est-à-dire une fréquence cardiaque supérieure ou égale à 100 bpm. L'explication possible c'est qu'à un moment donné, les chirurgiens ont effectué une infiltration péri-fistulaire d'une solution adrénalinée. Cette infiltration permet une vasoconstriction péri-fistulaire, distend le tissus cellulo-fibreux sous-cutané ou sous muqueux afin de favoriser la dissection. Le passage systémique de l'adrénaline ne faisait que limiter la survenue de la bradycardie et l'hypotension artérielle au prix d'une hypertension et d'une tachycardie. Nous avons aussi utilisé de la bupivacaïne isobare. En fait, les solutions isobares produisent une extension céphalique maximale moins importante et moins prévisible [14]. Il faut noter aussi que les pertes sanguines étaient minimes durant les interventions puisque la plupart des lésions de fistules obstétricales étaient classées simples et non compliquées dans notre série.

D'après l'étude de Reber et al, la position de lithotomie a peu de répercussion sur la mécanique respiratoire. Elle n'entraine pas de modification de la capacité résiduelle fonctionnelle (CRF) ni des rapports ventilation/perfusion [15]. Mais Lumb et al ont trouvé que la CRF pulmonaire est largement modifiée par la posture. La baisse de la CRF est d'environ 1L entre la position debout et le décubitus dorsal. La mise en position de lithotomie et de Trendelenburg aggrave cette amputation [16]. Dans la position de Trendelenburg, la pression exercée par les viscères abdominaux sur le diaphragme augmente, ce qui nécessite une augmentation des pressions de ventilation. Un bloc moteur trop étendu peut être à l'origine des difficultés respiratoires au cours de la rachianesthésie. En cas d'extension thoracique haute, la réduction de la participation intercostale à l'effort ventilatoire réduit le volume de réserve expiratoire et la capacité vitale [17]. La position de Trendelenburg pourrait majorer cette extension en fonction de la baricité du produit anesthésique [18]. Dans notre étude, l'état respiratoire varie en fonction du niveau anesthésique atteint, plus le niveau est supérieur et plus la fréquence respiratoire augmente (p=0,01). Par contre, ni l'âge, ni l'IMC, ni le test d'Emmel n'influent sur l'augmentation de la fréquence respiratoire. L'incidence de désaturation était nulle. Probablement à cause de la bupivacaïne isobare dont l'extension céphalique est moins importante, d'où la conséquence respiratoire quasiment moindre. En plus, notre population est jeune et nous avons choisi les patientes dépourvues de tare respiratoire pour l'étude. Notre étude a quand même des limites. A part le nombre faible de l'échantillon, nous avons juste réalisé une étude descriptive qui n'est pas très concluant pour concevoir une recommandation. La force de l'étude c'est qu'il s'agirait d'une toute première dans notre pays. Ainsi, nous avons l'intention d'approfondir les recherches. Le champ de l'étude est encore large et c'est encore un début, d'où la nécessité d'une collaboration entre les réanimateurs et les chirurgiens. D'autres études à grande échelle permettraient d'obtenir des résultats encore plus concluants.

## Conclusion

La chirurgie des fistules obstétricales est généralement réalisée sous rachianesthésie qui est la technique préférée car c'est simple, fiable, efficace et moindre coût. Les malades sont installées essentiellement en position de lithotomie exagérée avec une table d'opération inclinée en position de Trendelenburg pour que le chirurgien puisse facilement avoir accès au vagin. Certains auteurs ont rapporté quelques complications dues à cette posture avec parfois un arrêt cardio-respiratoire. Par contre, elle est bien supportée par nos patientes. Généralement, les paramètres hémodynamiques et respiratoires se trouvaient dans les limites physiologiques et nous n'avons marqué aucun incident d'arrêt cardio-respiratoire. Néanmoins, le plus important c'est d'éviter la survenue de cet événement, notamment lors des changements de position. Une étude avec un échantillon plus large ainsi qu'un protocole différent apporterait des résultats encore plus éloquents.

### Etat des connaissances actuelle sur le sujet

- L'opération de fistules obstétricales effectuée en position de lithotomie et Trendelenburg présente un risque théorique de survenue de difficultés hémodynamiques et respiratoires.

### Contribution de notre étude à la connaissance

- La consultation pré anesthésique est cruciale à la recherche des pathologies préexistantes, notamment cardio-vasculaires et respiratoires;
- L'utilisation de produits anesthésiques appropriés comme la bupivacaïne isobare adrénalinée (à extension céphalique moins importante), le monitorage et la remise en position de décubitus dorsal de façon progressive sont indispensables afin de limiter la survenue des complications.
